# Effects of different pretreatments on flavonoids and antioxidant activity of *Dryopteris erythrosora* leave

**DOI:** 10.1371/journal.pone.0200174

**Published:** 2019-01-02

**Authors:** Xinxin Zhang, Xin Wang, Minglong Wang, Jianguo Cao, Jianbo Xiao, Quanxi Wang

**Affiliations:** 1 College of Life and Environmental Sciences, Shanghai Normal University, Shanghai, China; 2 College of Life Science and Technology, Harbin Normal University, Harbin, China; 3 State Key Laboratory of Quality Research in Chinese Medicine, Institute of Chinese Medical Sciences, University of Macau, Taipa, Macau; 4 Shanghai Key Laboratory of Plant Functional Genomics and Resources, Chinese Academy of Sciences, Shanghai Chenshan Botanical Garden, Shanghai, China; Northeast Forestry University, CHINA

## Abstract

Flavonoids are secondary metabolites of plants that often have medical applications. The influences of different sample drying pretreatments on flavonoids and antioxidant activity of ferns have not studies. *Dryopteris erythrosora* leaves used to analyze flavonoid alterations resulting from drying pretreatments. The total flavonoid content of *D*. *erythrosora* leaves exposed to different pretreatments was significantly different. The total flavonoid content of samples initially air-dried in shade and then oven-dried at 75°C were the highest (7.6%), while samples initially dried at 75°C had the lowest content (2.17%). Antioxidant activities of *D*. *erythrosora* leaves with different pretreatments varied. Group B first air-dried in the shade and then oven-dried at 75°C and group C first air-dried in the sun and then oven-dried at 75°C, both showed relatively stronger antioxidant activity. The best pretreatment for preserving the flavonoids was to first dry the plant material in the shade and then complete the drying process in an oven at 75°C. It was tentatively identified 22 flavonoids among the four different pretreatments by HPLC-ESI-TOF-MS.

## Introduction

Flavonoids are plant secondary metabolites with medical applications [1]. Some factors influenced flavonoid levels, such as harvest time [2–4], shade netting, planting time [5], development [6], and using the light transmittance paper bags [7]. Sample processing can influence the quantity and quality of bioactive compounds [8–10]. For example, the flavonoid content of fresh mulberry leaves was highest and the content in leaves that were oven-dried at 100–105°C was lowest [11]. Flavonoid content, DPPH scavenging activity, and reducing power of *Salvia officinalis* L. leaves dried in the shade were higher than levels in leaves oven-dried at 65°C [12]. *Paramignya trimera* dried in an oven at 25°C had a higher flavonoid content than samples dried in an oven at 100°C [8]. Flavonoid yields differed in *Belamcanda chinensis* dried at temperatures ranging from 40°C to 120°C [13]. These studies demonstrated that drying conditions could alter the flavonoid content and biological activity of plant flavonoids.

However, the manner in which different drying pretreatments affect flavonoid levels remains unclear. Ferns generally have high flavonoid contents but there is little information on the effects of drying pretreatments on flavonoid levels. Therefore, *Dryopteris erythrosora* (Eaton) O. Ktze. (Dryopteridaceae) used for the analysis of flavonoids. The aims of this study were to: (I) assess the effects of different drying pretreatments on the flavonoids and the antioxidant activity of *D*. *erythrosora* leaves, and (II) determine the best drying pretreatment for conserving fern flavonoids and antioxidant activity.

## Materials and methods

### Plant materials

*D*. *erythrosora*, living in a partial shade habitat, were collected from Shanghai Sheshan National Forest Park in April 2017. The coordinates of the Shanghai Sheshan National Forest are E: 121°11'27.27"; N: 31° 5'47.41." The plants identified by Prof. Jianguo Cao. Voucher specimens deposited in the STC of the College of Life & Environmental Science, Shanghai Normal University.

### Chemicals

The chemicals used were the same as in a previous report [14]. Rutin (purity > 99.0%), 2,2-Diphenyl-1-picrylhydrazyl (DPPH), 2,2’-azinobis-(3-ethylbenzothiazoline-6-sulfonic acid) (ABTS), Nitrotetrazolium blue chloride (NBT), phenazine methosulfate (PMS), nicotinamide adenine dinucleotide (NADH), 5, 5’-dithiobis-(2-nitrobenzoic acid) (DTNB) and 2,4,6-tri-2-pyridyl-s-triazine (TPTZ) were purchased from Sigma Co. (Shanghai, China). Acetonitrile was purchased from Thermo Fisher Scientific (Shanghai, China).

### Preparation of plant extracts

Fresh leaves of *D*. *erythrosora* were randomly separated into four groups and then exposed to four treatments. Group A leaves cleaned and frozen in liquid nitrogen were pulverized. Group B leaves were initially dried in the shade about one day then oven-dried at 75°C for 48 h, before grinding them to a powder. Group C leaves were initially dried in the sun (on top of absorbent old newspapers), then oven-dried at 75°C for 48 h, then ground to a powder. Group D leaves were cleaned then initially oven-dried at 75°C for 48 h prior to grinding. Ground samples were passed through an 80-mesh screen.

Powders (1.0 g) from groups B, C, and D were separately added to 60% ethanol (25 mL) and disposed with ultrasound machine-assist (20 min) and then water-bath at 50°C for 2 h. The extraction process repeated twice, and the mixture was filtered using a vacuum suction filter pump with the solution volume maintained at 50 mL. To obtain the same dry weight, 3.3 g samples from group A ground in a mortar and then added to 60% ethanol (25 mL) for extraction. Extraction was prepared as the above.

One portion of the extract used for determining the total flavonoid content and antioxidant activity. The other portion was extracted by petroleum ether (PE), dichloromethane (DCM), ethyl acetate (EtOAc), and n-butanol (nBuOH) for HPLC-ESI-TOF-MS analysis of flavonoids.

### Determination of total flavonoids content

A colorimetric assay was used for determining flavonoid content. First, gradient concentration rutins were successively added to 5% NaNO_2_ for 6 min, 5% Al(NO_3_)_3_ for 6 min, 4% NaOH for 12 min, and then the optical density (OD) of the mixture was recorded at 510 nm. Second, the linear equation (y = A+Bx) of rutin was plotted using Origin 7.5 software. Third, we determined the optical density (OD) of the gradient concentration extracts. The total flavonoid content was calculated as follows: total flavonoid content (%) = [(OD_1_+OD_2_+OD_3_)/3-A]/B*10/2*Volume/1000*100% [14].

### Antioxidant activity

#### DPPH assay

The DPPH free radical scavenging activity assay was based on information in a previous study [14]. Briefly, 1 mL DPPH (0.1 mM in ethanol) and extracts with a gradient of concentrations were mixed. After incubation for 30 min, the absorbance value was measured at 517 nm. A 60% methanol sample was used as the control group. The DPPH free radical scavenging activity was calculated using the following formula: DPPH free radical scavenging activity (%) = (1-A_sample 517_/A_control 517_)*100. The experiments were performed in triplicate (RSD < 5.0%).

#### ABTS assay

The ABTS assay of the extracts was previously described [14]. Briefly, 150 μL extracts with gradient concentration and 3 mL of appropriately diluted ABTS solutions were mixed. After incubating for 6 min, the absorbance value at 734 nm was determined. The ABTS free radical scavenging activity was calculated by: ABTS free radical scavenging activity (%) = (1-A_sample 734_/A_control 734_)*100. The experiments were performed in triplicate (RSD < 5.0%).

#### Superoxide anion (O^2-^) scavenging activity

Determination of superoxide anion (O^2-^) scavenging activity was the same as described in a previous study [14]. In brief, 1 mL extracts with gradient concentrations were mixed with sodium phosphate buffer and added to 1 mL NBT (150 μM), 1mL NADH (468 μM), and 1 mL PMS (60 μM) in turns, incubating at 25°C for 5 min. The absorbance at 560 nm was then determined. Superoxide anion (O^2-^) scavenging activity was calculated by: Superoxide anion (O^2-^) scavenging activity (%) = (1-A_sample 560_/A_control 560_)*100. The experiments were performed in triplicate (RSD < 5.0%).

#### Reducing power assay

The reducing power assay was reported previously [14]. The mixture, which included 1 mL extract with gradient concentration, 2.5 mL phosphate buffer, and 2.5 mL potassium ferricyanide, was maintained in a water bath at 50°C for 20 min, then 10% TCA was added to terminate the reaction. After centrifugation, 50% of the supernatant was mixed with 2.5 mL of distilled water and 0.5 mL of 0.1% ferric chloride. The remaining 50% of the supernatant was mixed with 3 mL of distilled water as the control group. Optical density at 700 nm reflected the reducing power. The experiments were performed in triplicate with similar results (RSD < 5.0%).

#### FRAP assay

The FRAP assay was described in a previous report [14]. The FRAP reagent was made up with TPTZ (10 mM) in HCl solution (40 mM) and FeCl_3_ (20 mM) in 250 mL acetate buffer (pH 3.6). The FRAP reagent was used immediately after preparation. A gradient concentration of extracts was added to the FRAP reagent. After 4 min, the optical density of the mixture at 593 nm was determined. The calibration curves of Fe^2+^ were used to calculate the results. The FRAP reagent with distilled water was used as the control group. The experiments were performed in triplicate with similar results (RSD < 5.0%).

### Flavonoid analysis of *D*. *erythrosora* leaves with different drying pretreatments using HPLC-ESI-TOF-MS

Chromatographic separation was performed on an Agilent 1100 HPLC system (USA Agilent Technologies), equipped with a binary pump, a microdegasser, Hi-performance well-plate auto sampler, thermostat column compartment, and diode-array detector (DAD). UV spectra were recorded between 190 and 400 nm, and the UV detector was set at 254 nm. Separation was performed on a SHISEIDO MG-C18 (1003.3 mm; i.d. 3.0 mm) column using a gradient elution [methanol (A)/ water (0.1%HCOOH)(B)].

Extractions of petroleum ether (PE), dichloromethane (DCM), ethyl acetate (EtOAc), and n-butanol (nBuOH) were each diluted 10 times. The gradient program was 0–15 min, 15–45% A; 15–25 min, 45–55% A; 25–35 min, 55–90% A; the flow rate maintained at 0.4 mL/min, and the sample injection volume was 10 μL. The column temperature was set at 25°C. All of the MS experiments conducted on an Agilent 6220 Time-of-Flight mass spectrometer (TOF) equipped with an electrospray ionization (ESI) interface (Agilent Technologies, USA). Both the auxiliary and nebulizer gases were nitrogen with flow rates of 10 L/min. The MS analysis was performed in both positive and negative scan modes under the following operation parameters: dry gas temperature = 350°C, voltage = 160V and the nebulizer pressure = 45 psi. Full scan data acquisition and dependent scan event data acquisition were performed from m/z 100–1200.

## Results and discussion

### Total flavonoid content of *D*. *erythrosora* leaves with different drying pretreatments

**Fig 1:**
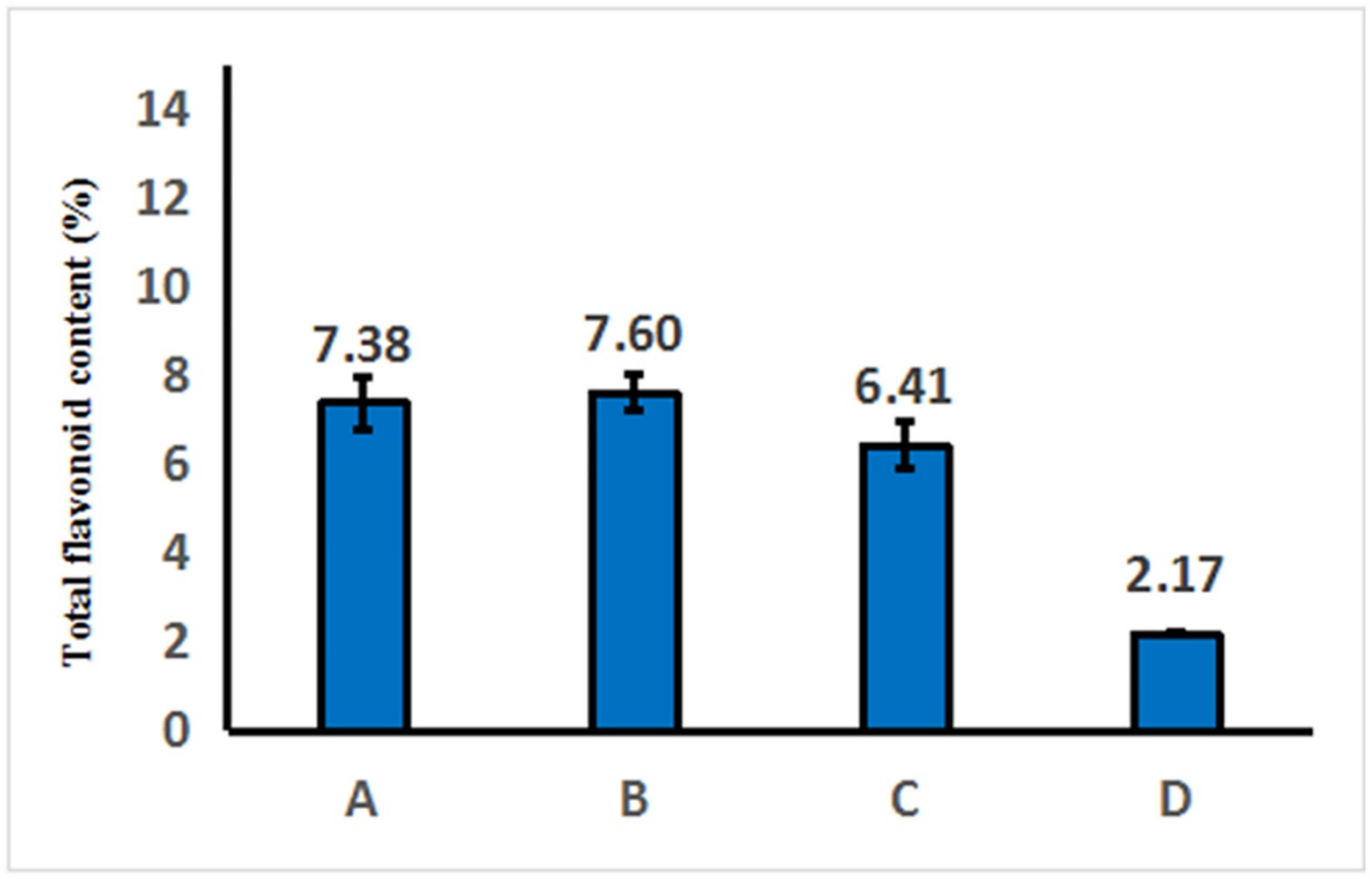
Total flavonoid content of *D*. *erythrosora* leaves with different drying pretreatments. Group A: liquid nitrogen freezing-grinding directly; Group B: shade-drying directly; Group C: sun-drying directly; Group D: direct heating at 75°C.

Datas from the standard curve of Rutin were list in S1 Table. The total flavonoid contents of *D*. *erythrosora* leaves with different drying pretreatments were 7.38% (Group A), 7.6% (Group B), 6.41% (Group C), and 2.17% (Group D), respectively (Fig 1). The total flavonoid content of extracts from group B, which were first dried about one day then completely oven-dried at 75°C was the highest. The flavonoid content from group D, dried at 75°C in oven directly after cleaning, was the lowest.

The process of drying in the shade produced a slow rate of water loss.which might result in the increasing of total flavonoid content. Sun-drying produced a faster rate of water loss, ending flavonoid metabolism. Full sunlight exposure can influence flavonoid metabolites in leaves [15–16], which results in a decreased flavonoid content. Samples from group A, which were ground with liquid nitrogen, showed the total flavonoid content similar to the live samples. We speculate that water and sunlight are the main factors affecting total flavonoid content in leaf samples. In addition, flavonoids are heat sensitive [17]. Heating at 75°C directly can destroy enzyme activity and block the synthesis pathway of flavonoids. This may be the reason why the total flavonoid content of Group D was the lowest.

### Antioxidant activity

**Fig 2:**
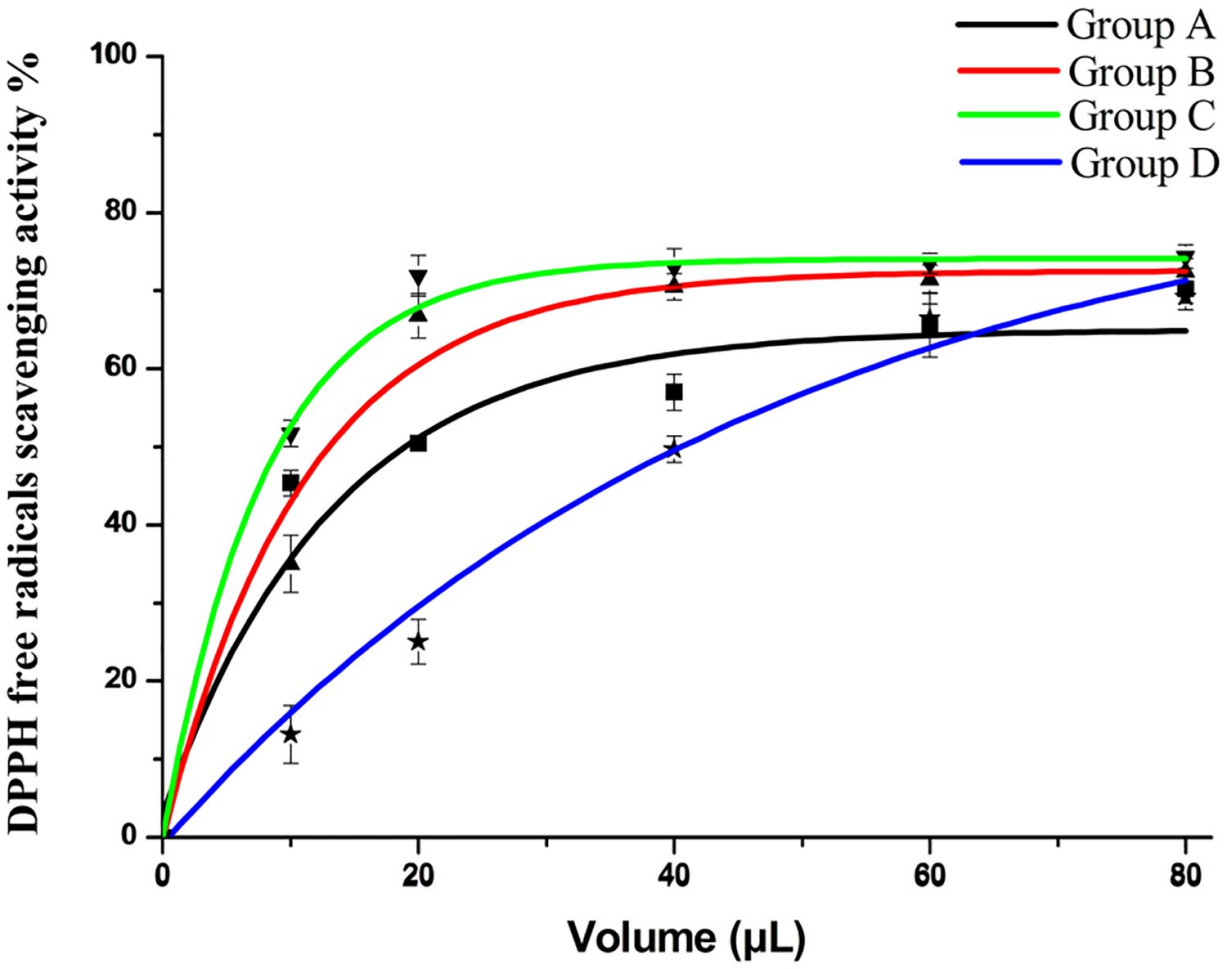
DPPH free radicals scavenging activity of *D*. *erythrosora* leaves with different drying pretreatments. Group A: liquid nitrogen freezing-grinding directly; Group B: shade-drying directly; Group C: sun-drying directly; Group D: heating at 75°C directly.

The DPPH free radical scavenging activities of *D*. *erythrosora* leaf extracts with different pretreatments were shown in Fig 2. When the doses were from 0–80 μL, the DPPH free radical scavenging potential increased. Group C was higher than group B. Fewer than 10 μL of the extracts could scavenge about 50% of the free radicals. However, group D was significantly lower than group C. The IC 50 of groups A, B, C, and D were 0.6 μg/mL, 0.38 μg/mL, 0.25 μg/mL, and 0.35 μg/mL, respectively. The DPPH free radical scavenging activity of the extracts was arranged as group C>group B>group A>group D.

**Fig 3:**
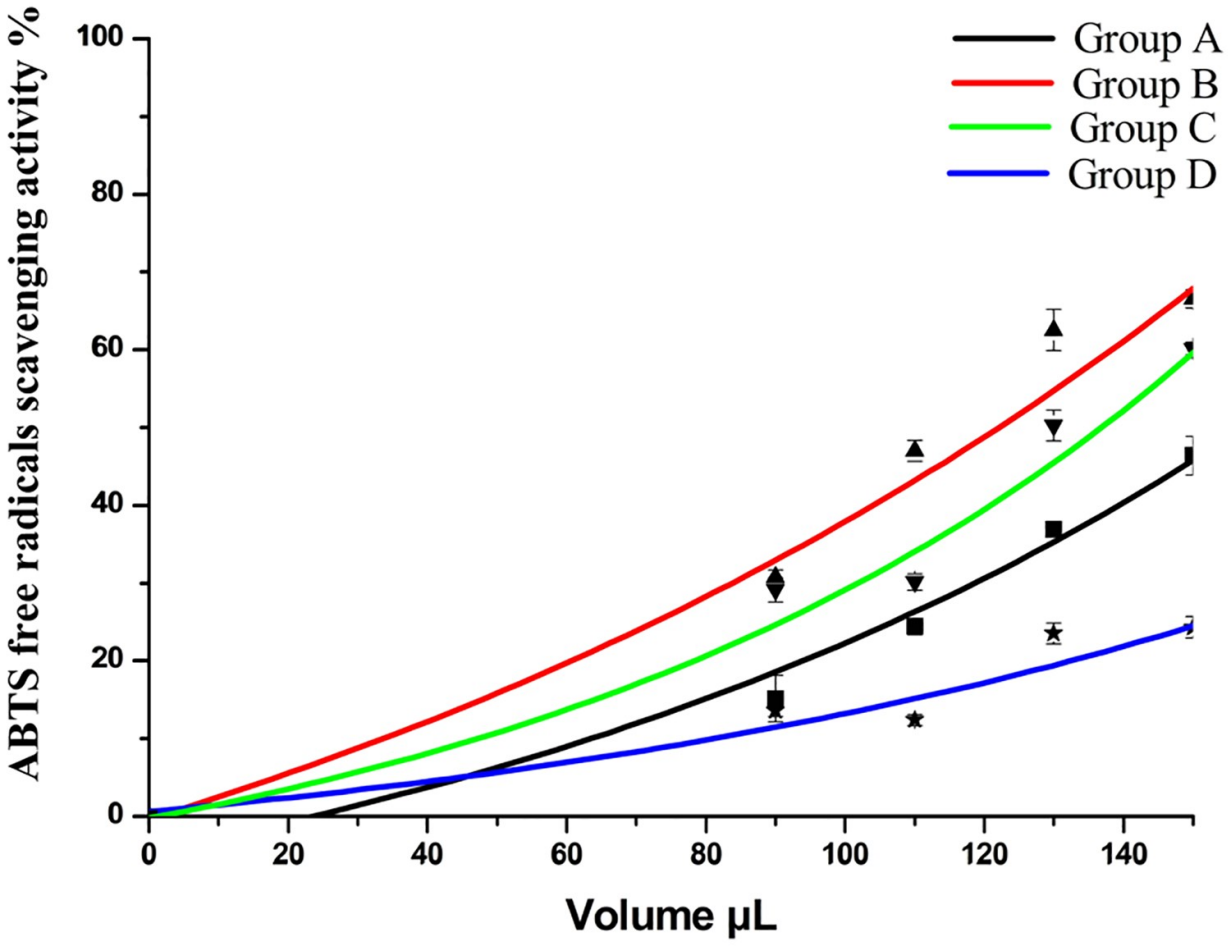
ABTS free radicals scavenging activity of *D*. *erythrosora* leaves with different drying pretreatments. Group A: liquid nitrogen freezing-grinding directly; Group B: shade-drying directly; Group C: sun-drying directly; Group D: heating at 75°C directly.

The ABTS free radical scavenging activities are shown in Fig 3. When the volume was between 0–150 μL, the ABTS free radical scavenging potential increased. Group B was the highest and group D the lowest. A 120 μL amount of the extract scavenged about 50% of the free radicals. The IC 50 of groups A, B, and C were 4.62 μg/mL, 3.72 μg/mL, and 3.52 μg/mL, respectively. The ABTS free radical scavenging potential of the groups were: B> C> A> D

**Fig 4:**
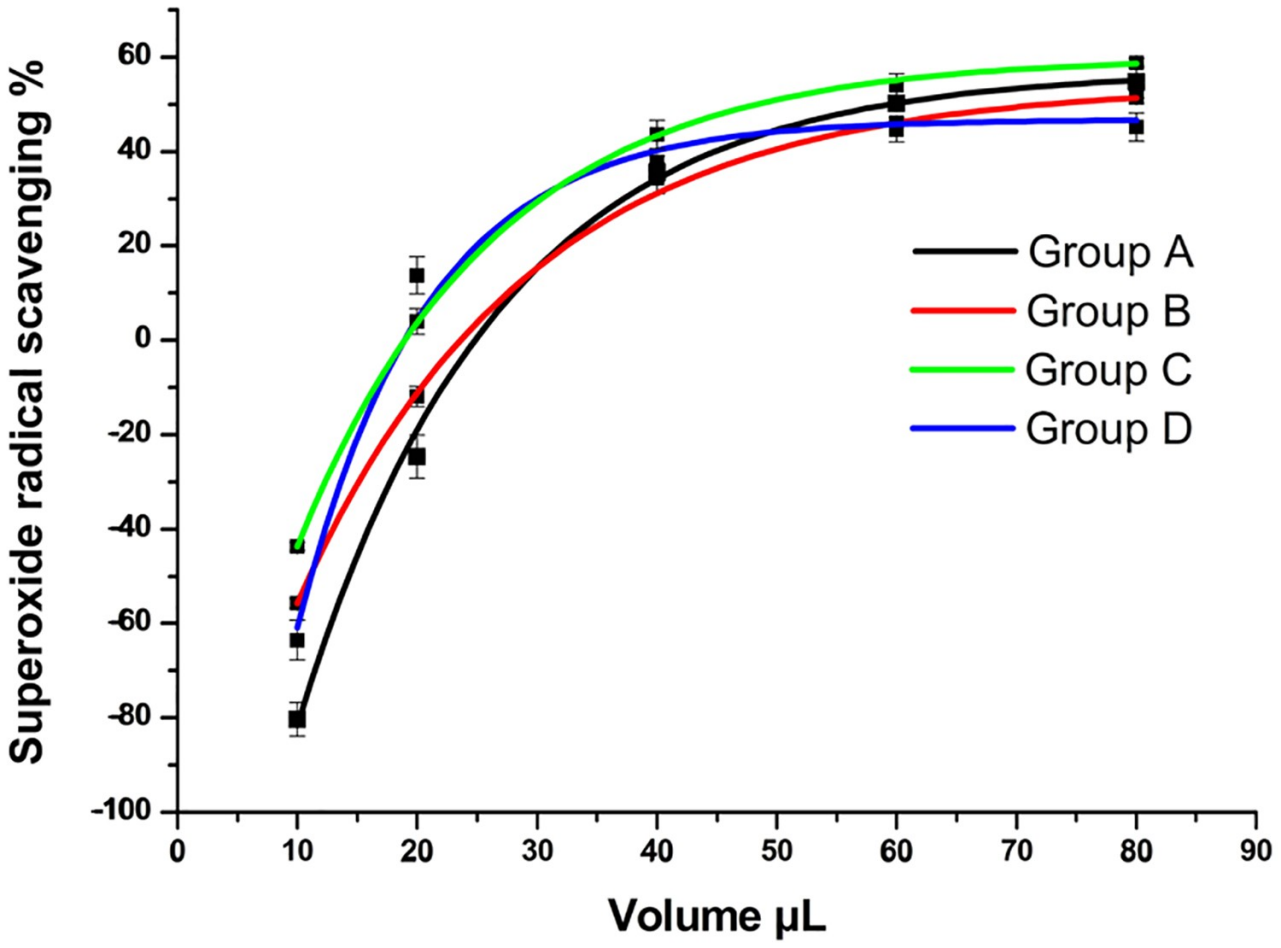
Superoxide radicals scavenging activity of *D*. *erythrosora* leaves with different drying pretreatments. Group A: liquid nitrogen freezing-grinding directly; Group B: shade-drying directly; Group C: sun-drying directly; Group D: heating at 75°C directly.

The superoxide anion scavenging activity showed in Fig 4. In the range from 10–90 μL, the superoxide anion scavenging potential increased. The samples that were first dried in the sun and then oven-dried at 75°C had relatively stronger superoxide scavenging activities. The IC 50 values of groups A, B, and C were 1.81 μg/mL, 2.28 μg/mL, and 1.25 μg/mL, respectively.

**Fig 5:**
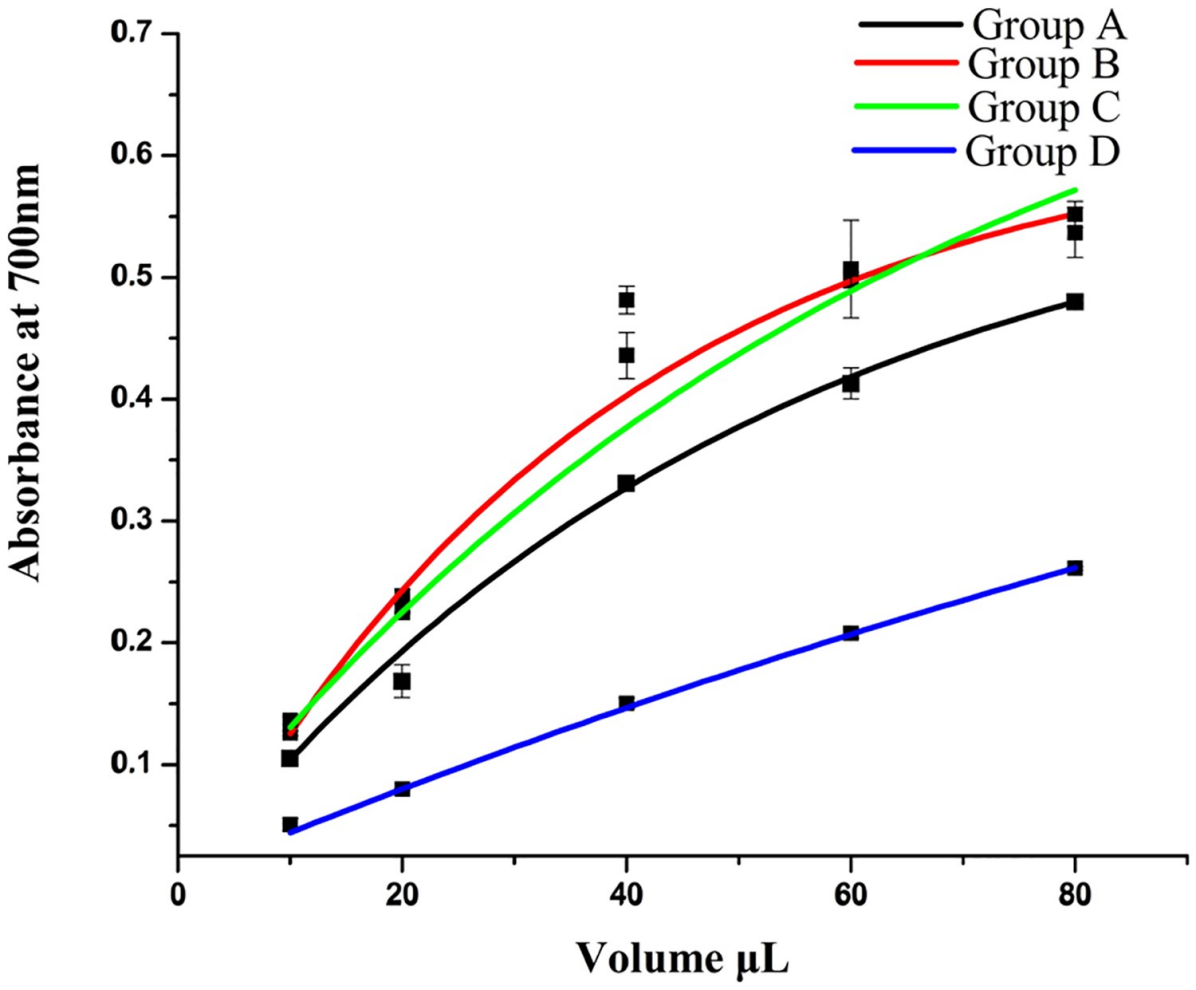
Reducing power of *D*. *erythrosora* leaves with different drying pretreatments. Group A: liquid nitrogen freezing-grinding directly; Group B: shade-drying directly; Group C: sun-drying directly; Group D: heating at 75°C directly.

**Fig 6:**
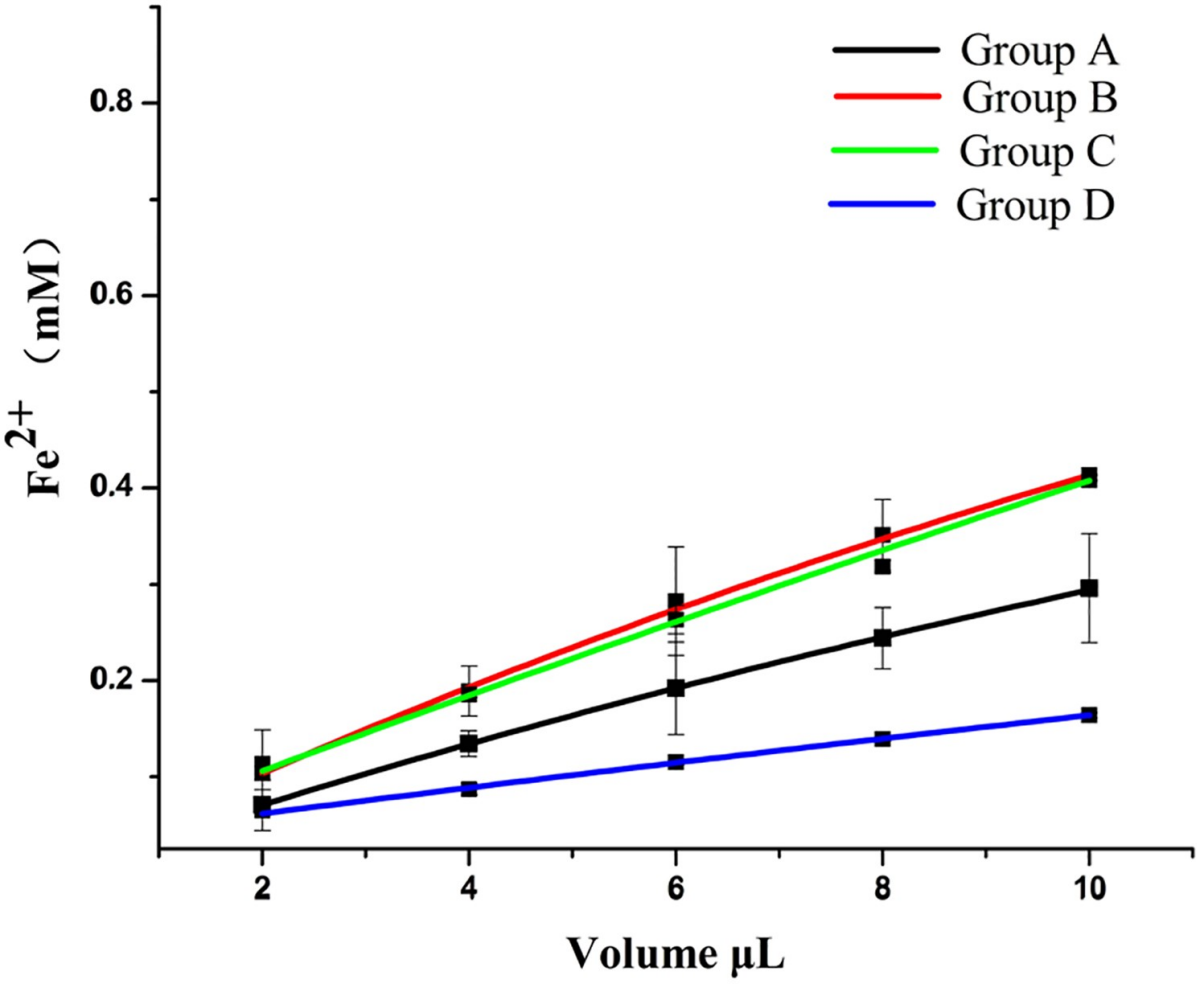
The FRAP of *D*. *erythrosora* leaves with different drying pretreatments. Group A: liquid nitrogen freezing-grinding directly; Group B: shade-drying directly; Group C: sun-drying directly; Group D: heating at 75°C directly.

The FRAP assay and reducing power assay results are shown in Figs 5 and 6. Extracts from *D*. *erythrosora* leaves with different drying pretreatments possessed both antioxidant and reductive activity of Fe^3+^. With increased volume, the activity increased. The activity level between groups was: B> C> A> D.

These results illustrated that different drying pretreatments altered the antioxidant activities of *D*. *erythrosora* leaves. Except for the superoxide anion (O^2-^) scavenging assay, groups B and C both had stronger antioxidant activity than group D, which might be related to the flavonoid contents.

### Flavonoids analysis of *D*. *erythrosora* leaves with different drying pretreatments using HPLC-ESI-TOF-MS

HPLC-ESI-TOF-MS was used for the qualitative analysis of flavonoids of *D*. *erythrosora* leaves. Comparing the data with known chromatograms and mass spectral data, a total of 22 peaks were tentatively identified as flavonoids (Table 1). There were 8 flavonols, 4 flavones, 3 chalcones, 2 flavanols, 2 flavanones, 1 homoisoflavones, 1 isoflavone, and 1 isoflavanone in the mass spectrometry-total ions chromatogram (MS-TIC) of extracts from *D*. *erythrosora* leaves with different drying pretreatments in the negative ion mode. The main flavonoids of *D*. *erythrosora* leaves were flavone and flavonols, and this result is consistent with previous studies [18–22].

**Table 1:** Analysis of *D*. *erythrosora* leaves with different pretreatments by HPLC-ESI-TOF-MS.

No	Rt	Formula	M/Dalton	M/MFG	m/z [M-H]^-^	ppm	UV/nm	Identification	Compound type	A ×10^7^	B ×10^7^	C ×10^7^	D ×10^7^	Ref.
1.	15.53^a^	C17H16O8	348.0847	348.0845	347.0774	-0.46	245, 280	(+)-catechin-8-acetic acid	flavanol	0.14	1.61	ND	ND	[27]
2.	19.00^a^	C20H18O11	434.086	434.0849	433.0793	-2.45	245, 270	Quercetin-3-O-β-D-xylopyranoside	flavonol	2.00	1.32	0.21	ND	[28]
3.	19.37^a^	C21H20O13	480.0903	480.09	479.0831	-0.82	245, 270	Myricetin 3-O-glucoside	flavonol	0.19	1.95	0.73	ND	[29]
4.	20.35^a^	C27H30O15	594.1591	594.1585	593.1518	-1.05	270, 287	Scutellarein 7-O-glucobioside	flavone	0.15	1.05	0.29	1.68	[30]
5.	21.97^a^	C20H30O16	610.1541	610.1534	609.1468	-1.18	258, 355	Rutin	flavonol	2.23	7.11	1.22	ND	[31]
6.	22.79^a^	C27H30O14	578.1632	578.1636	577.1559	0.62	245, 268	Apigenin 7-O-rutinoside	flavone	4.67	3.54	4.02	0.73	[32]
7.	26.96^a^	C20H18O9	402.0957	402.0951	401.0885	-1.61	255, 320	Apigenin-C-pentoside	flavone	4.08	11.96	8.05	0.69	[33]
8.	28.26^a^	C17H18O7	334.1058	334.1053	333.098	-1.61	255, 280	10-O-Methylhematoxylol B/10-O-methylepihematoxylol B	Homoisoflavonoid	1.03	0.27	ND	0.24	[34]
9.	29.27^a^	C26H26O11	514.1476	514.1475	513.1402	-0.21	255, 280	Koreanoside B	flavonol	1.03	1.10	0.72	ND	[35]
10.	12.50^b^	C32H36O18	708.1898	707.1902	707.1822	0.9	240, 295, 330	Kaempferide 3-Rhamnoside-7-(6"-Succinylglucose)	flavonol	19.40	42.74	21.25	ND	[36]
11.	17.95^b^	C26H28O14	564.1487	564.1479	563.1414	-1.35	255, 335	Apigenin-6-C-ara-8-C-glu	flavone	0.90	0.56	0.28	ND	[37]
12.	22.69^b^	C21H20O12	464.0962	464.0955	463.0889	-1.54	255, 355	Isocarthamidin-7-O-glucuronide	flavonol	0.87	2.01	2.13	ND	[38,39]
13.	24.50^b^	C20H18O10	418.0896	418.09	417.0823	0.95	245, 330	Kaempferol 3-O-α-L-arabinopyranoside	flavonol	18.14	3.62	4.98	0.39	[40]
14.	30.35^c^	C30H30O15	630.159	630.1585	629.1517	-0.82	250, 270	2S-5,7,2',5'-Tetrahydroxy-6-methoxyflavanone	flavanone	ND	1.00	0.26	ND	[41]
15.	37.80^c^	C22H26O9	434.1581	434.1577	433.1508	-0.89	250, 285	2'-Hydroxy-2,3,4,5,4',5',6'-heptamethoxychalcone	chalcone	ND	4.68	ND	ND	[42]
16.	49.55^c^	C23H28O9	448.1723	448.1733	447.1660	2.31	250	2,3,4,5,2',4',5',6'-Octamethoxychalcone	chalcone	ND	5.65	ND	1.37	[43]
17.	57.21^c^	C30H34O11	570.2096	570.2101	569.2023	0.9	250	(-)-5,7-O-Dimethyl-3′,4′,5′-O-trimethylepigallocatechin-3-O-(3′′,4′′,5′′-O-trimethyl) gallate	flavanol	ND	2.84	ND	ND	[44]
18.	26.18^d^	C25H24O12	516.127	516.1268	515.1198	-0.5	260, 330	Formononetin-7-O-(6''-malonylglucoside)	isoflavone	ND	0.47	ND	ND	[45]
19.	34.73^d^	C24H26O13	522.1375	522.1373	521.1302	-0.35	255, 280	(2S)-5-Hydroxy-7,8,6'-trimethoxyflavanone-2'-O-b-D-glucuronide	flavanone	ND	0.86	ND	ND	[46,47]
20.	37.43^d^	C26H30O13	550.1689	550.1686	549.1616	-0.51	255	Isoliquiritin apioside	chalcone	ND	0.46	0.20	ND	[48,49]
21.	16.35^e^	C25H24O13	532.122	532.1217	531.1147	-0.51	243, 285	Biochanin A-7-O-glucoside-6”-O-malonate	isoflavanone	ND	4.08	0.31	ND	[50]
22.	19.9^e^	C27H30O17	626.1493	626.1483	625.1421	-1.64	245, 270	Quercetin-O-dihexoside	flavonol	ND	0.84	0.38	ND	[51]

^a^: total ion chromatograms of ethyl acetate of extracts from group A;

^b^: total ion chromatograms of n-butanol of extracts from group A;

^c^: total ion chromatograms of dichloromethane of extracts from group B;

^d^: total ion chromatograms of ethyl acetate of extracts from group B;

^e^: total ion chromatograms of n-butanol of extracts from group B

Chalcones and isoflavones were absent in group A but present in groups B and C. This is the first report of these compounds in the Dryopteridaceae. This result showed chalcones and isoflavones could not be synthetized during natural growth of *D*. *erythrosora* leaves but could result from the stresses of sun-drying or shade-drying. Except for anthocyanin and its derivatives, all of the flavonoid types in the flavonoid synthesis pathway could be found [23]. We concluded that the flavonoid metabolic pathways of ferns are similar to spermatophytes and that the metabolic pathways are closely related to the stress response.

**Fig 7:**
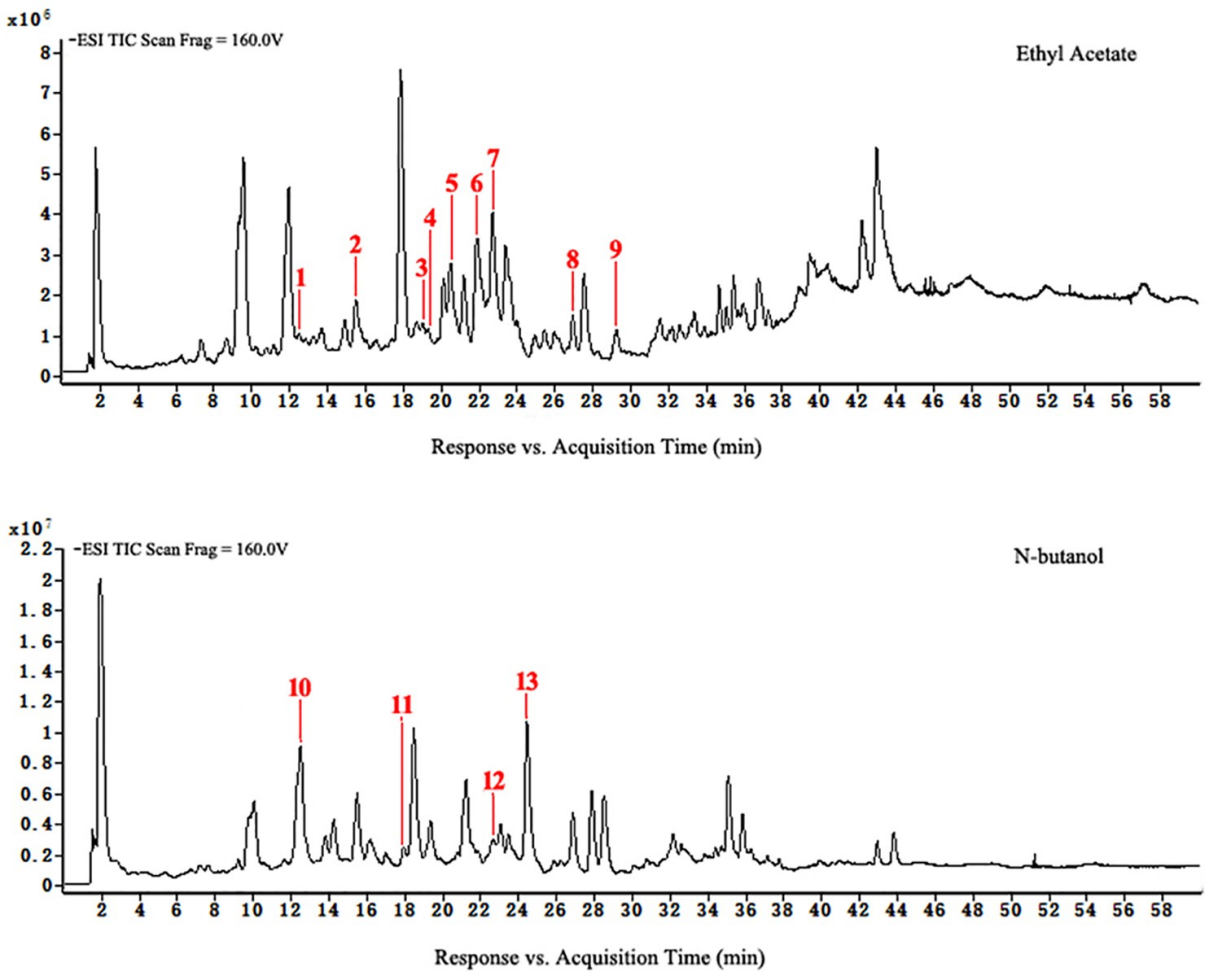
HPLC-ESI-TOF-MS total ion chromatograms of ethyl acetate and n-butanol of extracts from group A.

**Fig 8:**
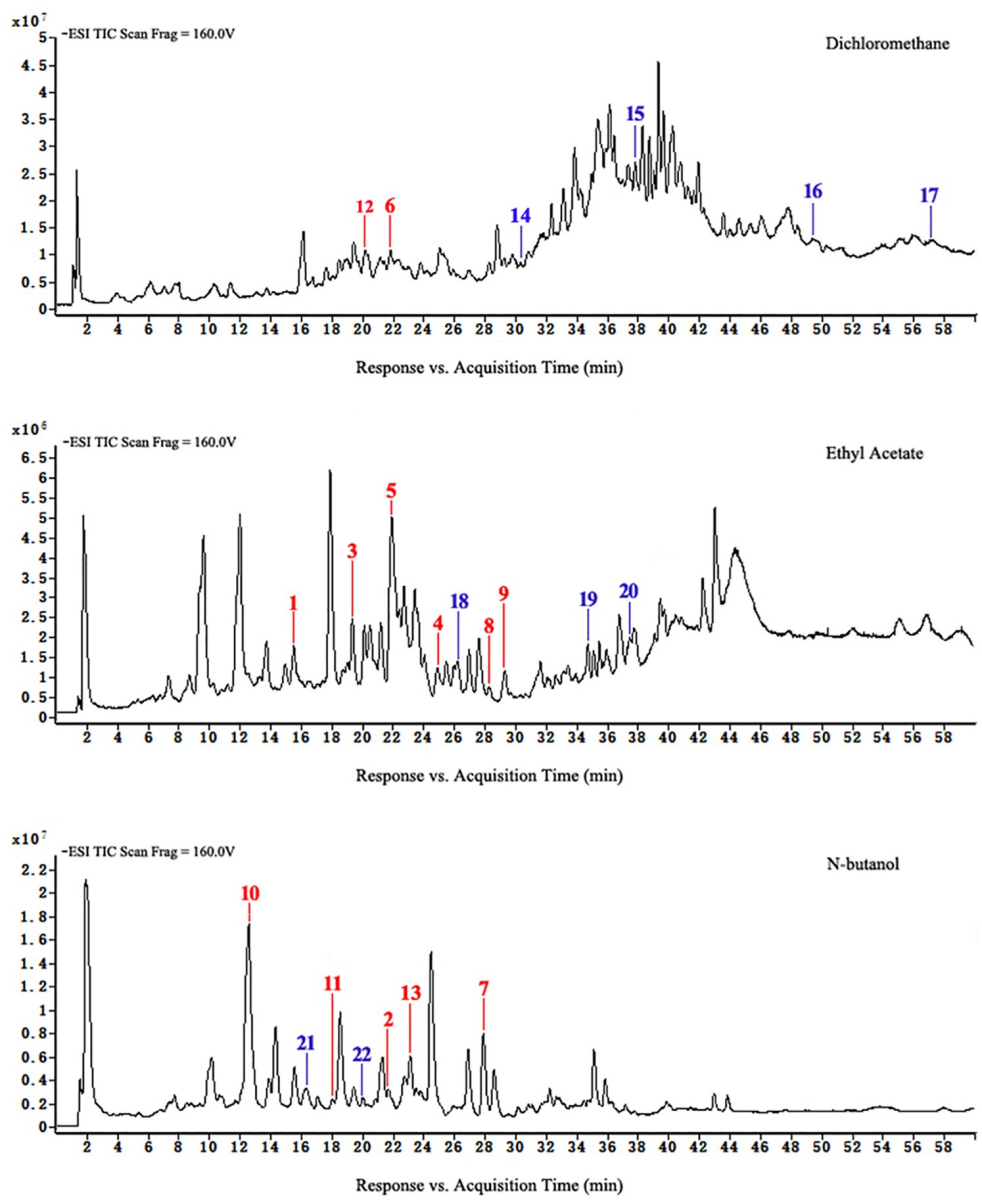
HPLC-ESI-TOF-MS total ion chromatograms of dichloromethane, ethyl acetate, and n-butanol of extracts from group B.

**Fig 9:**
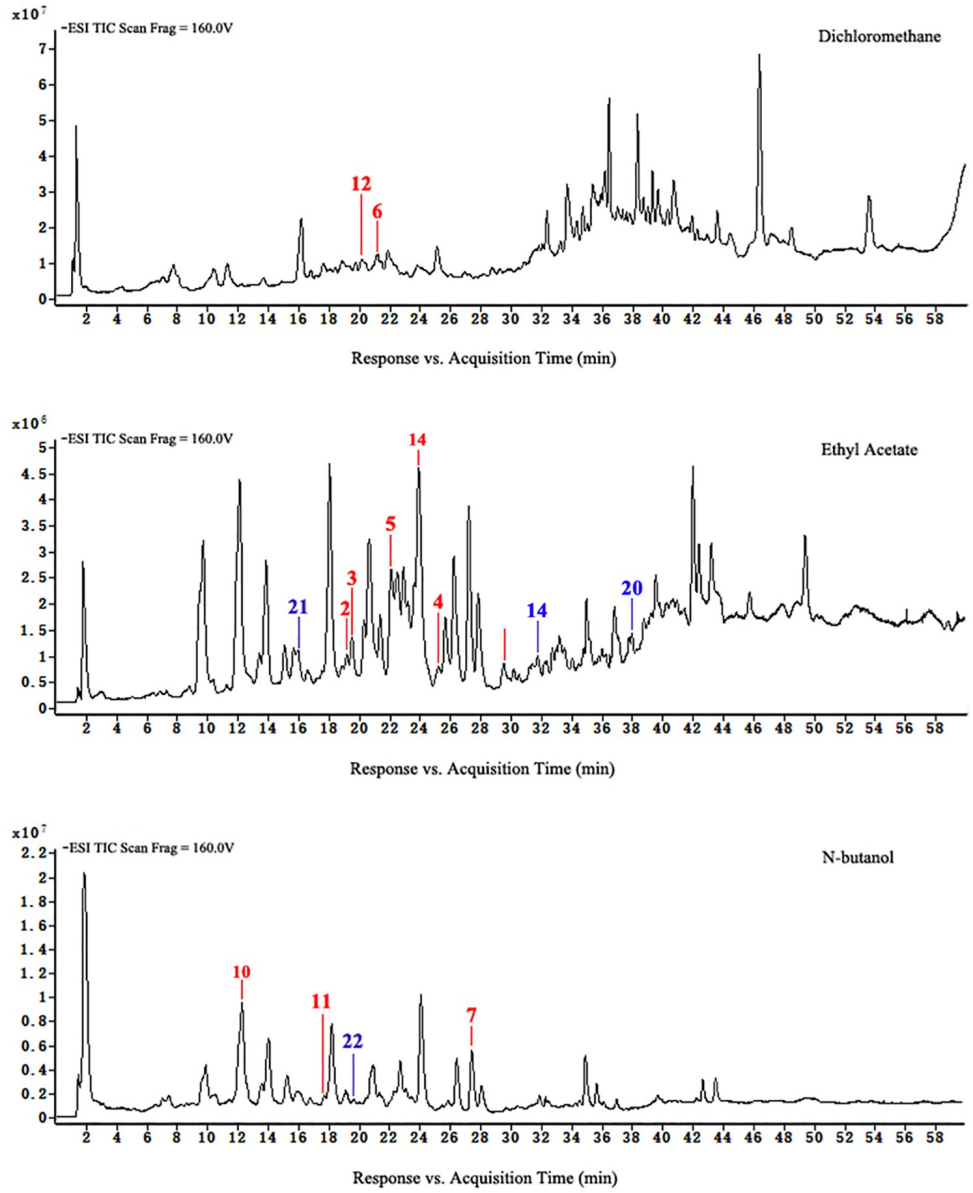
HPLC-ESI-TOF-MS total ion chromatograms of dichloromethane, ethyl acetate, and n-butanol of extracts from group C.

**Fig 10:**
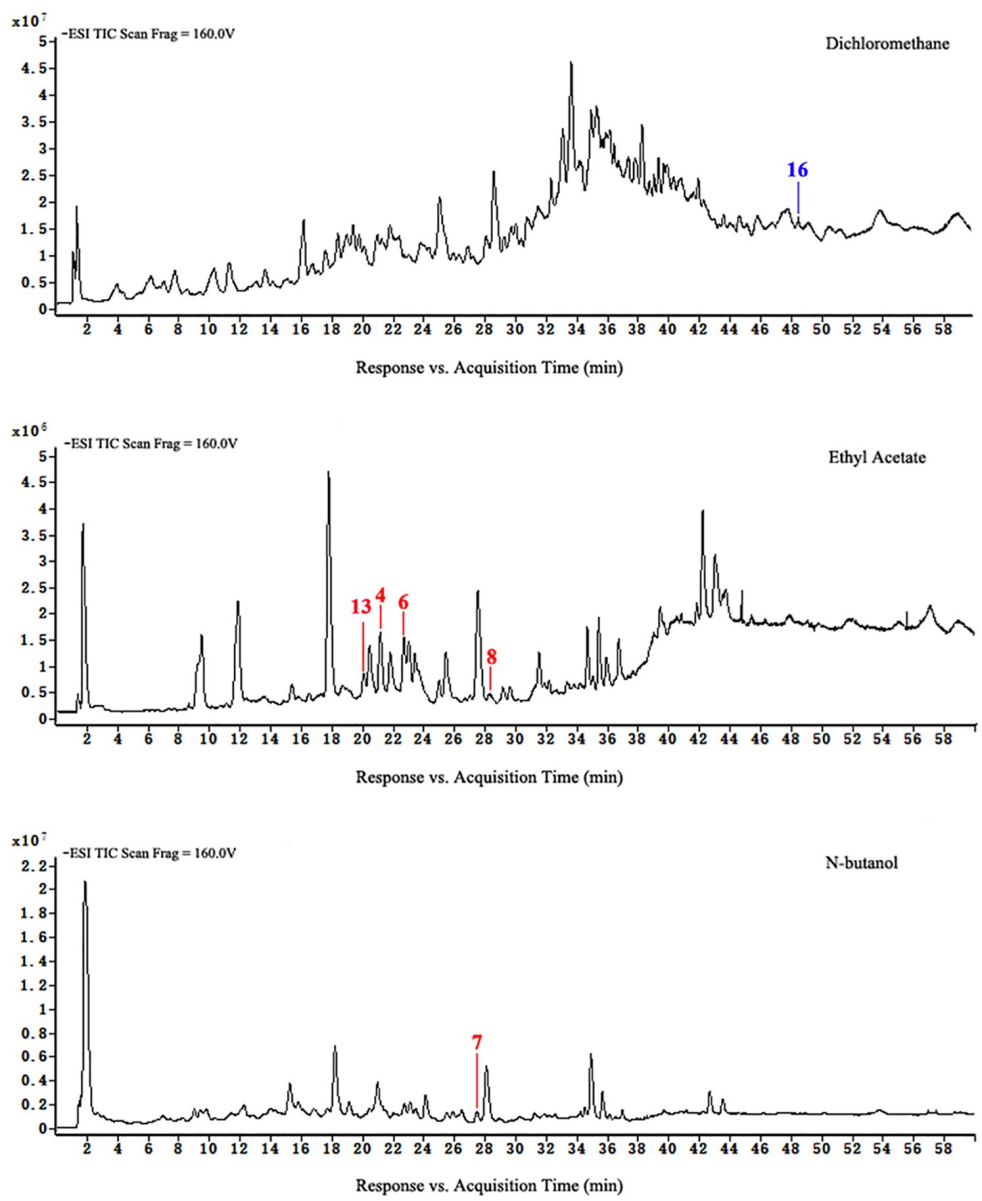
HPLC-ESI-TOF-MS total ion chromatograms of dichloromethane, ethyl acetate, and n-butanol of extracts from group D.

**Fig 11:**
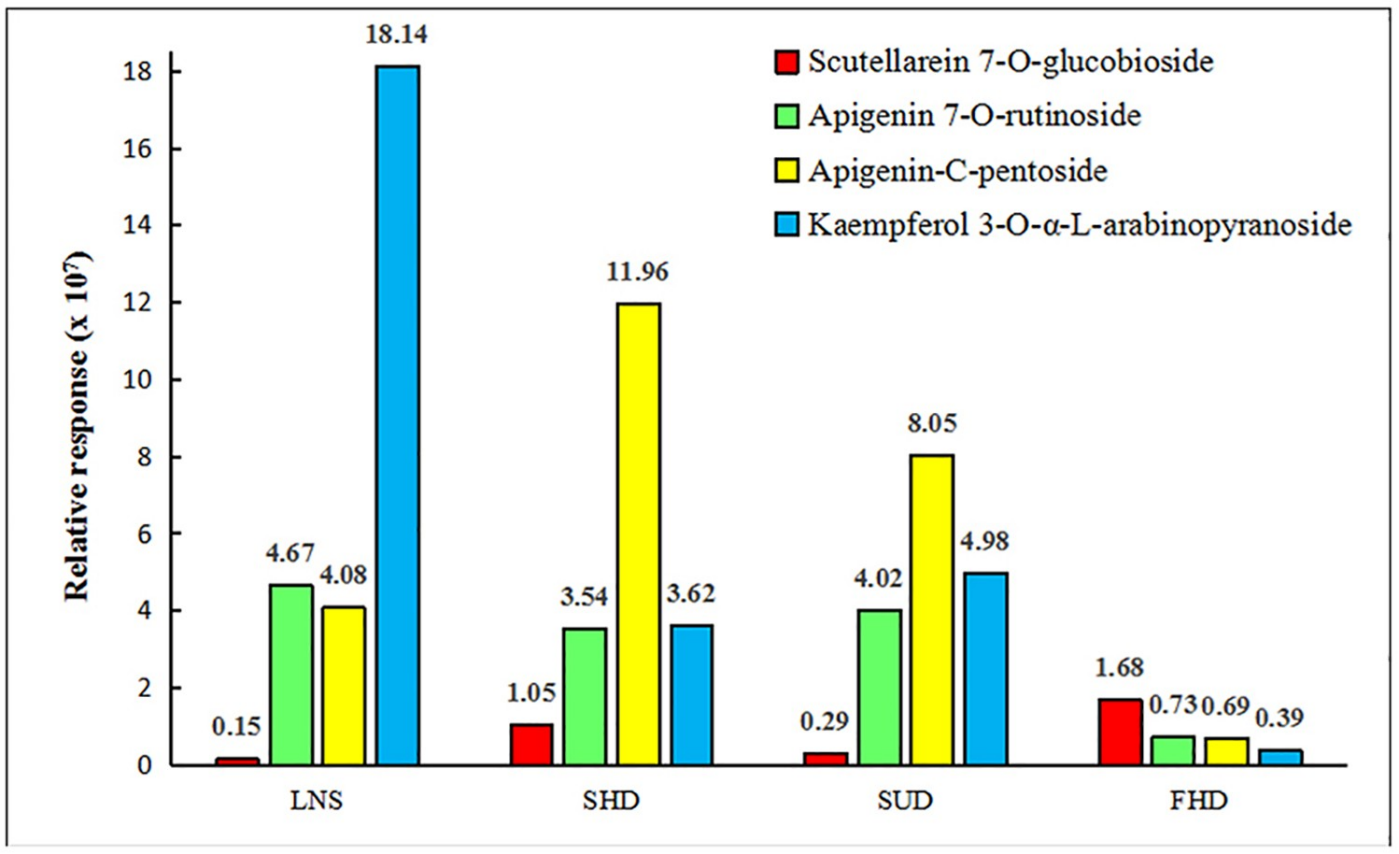
The common flavonoids with four different drying pretreatments (LNS: Group A; SHD: Group B; SUD: Group C; and FHD: Group D).

Loss of flavonoids from group D was the greatest. Figs 7 to 10 show that scutellarein 7-O-glucobioside, apigenin 7-O-rutinoside, apigenin-C-pentoside, and kaempferol 3-O-α-L-arabinopyranoside are common flavonoids. This suggests that the flavonoid biosynthesis of the four components was unaffected by the drying pretreatments. However, the contents of the four flavonoids significantly changed (Fig 11). We used Group A samples (frozen-ground in liquid nitrogen) as the standard and found that the content of scutellarein 7-O-glucobioside increased. The largest amounts of these compounds were from group D. This result indicated that heating directly accelerated the synthesis of scutellarein 7-O-glucobioside.

The contents of apigenin 7-O-rutinoside and kaempferol 3-O-α-L-arabinopyranoside both decreased, and the lowest content was in group D. This demonstrated that the two compounds might decompose or transformed with direct heating. In addition, the amounts of apigenin-C-pentoside of groups B and C increased, but decreased in group D. This suggests that temperature might be the main factor influencing the synthesis of apigenin-C-pentoside.

**Fig 12:**
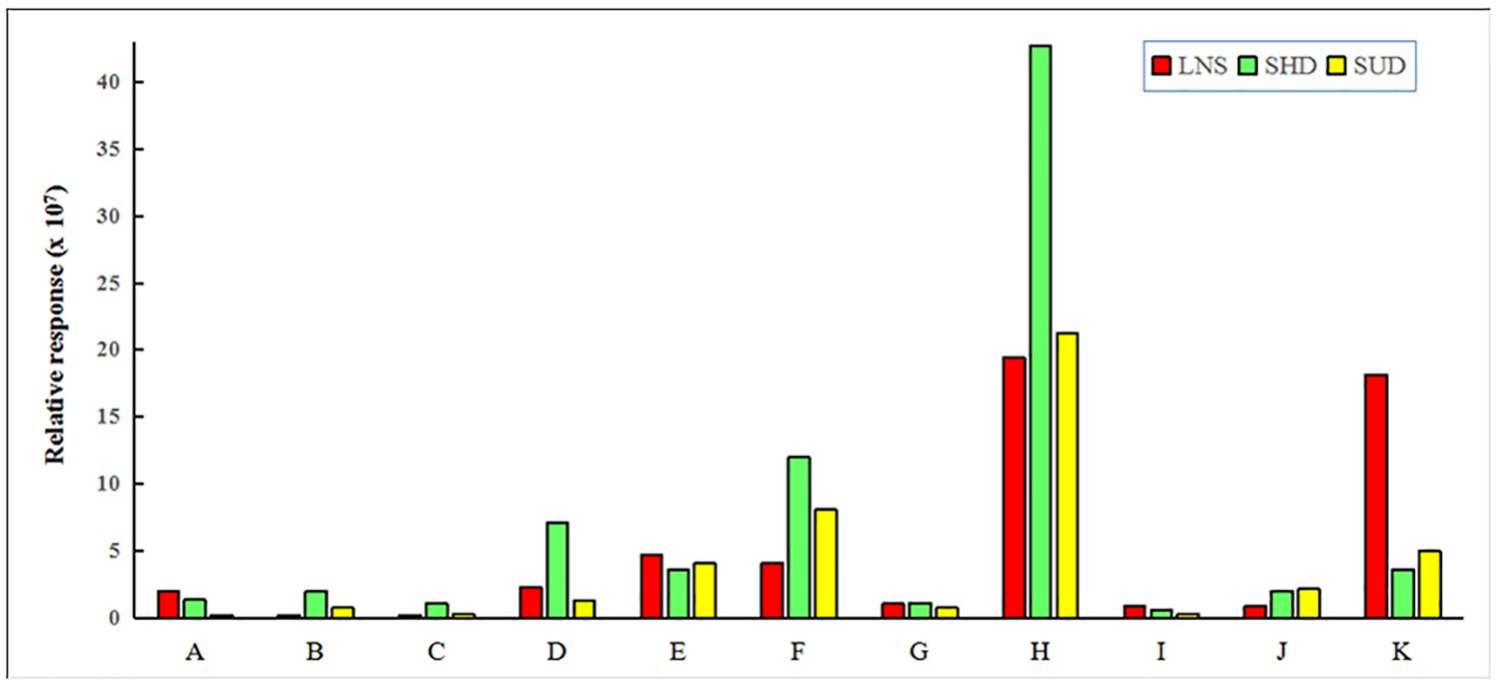
The common flavonoids with three different drying prtreatments (LNS: Group A; SHD: Group B; and SUD: Group C). A: Quercetin-3-O-β-D-xylopyranoside; B: Myricetin 3-O-glucoside; C: Scutellarein 7-O-glucobioside; D: Rutin; E: Apigenin 7-O-rutinoside; F: Apigenin-C-pentoside; G: Koreanoside B; H: Kaempferide 3-Rhamnoside-7-(6"-Succinylglucose); I: Apigenin-6-C-ara-8-C-glu; J: Isocarthamidin-7-O-glucuronide; K: Kaempferol 3-O-α-L-arabinopyranoside.

Among groups A, B, and C, 11 common flavonoids were found. The contents of these flavonoids also changed (Fig 12). The key difference in the drying pretreatments between groups B and C was the dehydration rate, which resulted in a myricetin 3-O-glucoside, kaempferide 3-rhamnoside-7-(6"-succinylglucose), and isocarthamidin-7-O-glucuronide increase but a reduction in quercetin-3-O-β-D-xylopyranoside and apigenin-6-C-ara-8-C-glu. The content of koreanoside B changed little, indicating that the synthesis of koreanoside B was not influenced. The decreased rutin may have resulted from the decomposition or the transformation.

The number of flavonoids was greatest in group B. Compared to group A, group B had 9 additional flavonoids and the contents of 8 common compounds increased. The levels of kaempferide 3-rhamnoside-7-(6"-succinylglucose) were 2× greater than group A.

High light irradiance can influence the biosynthesis of dihydroxy B-ring-substituted flavonoids [24–26]. In this study, the effects of light irradiance were not obvious. Among the different drying pretreaments, the loss of flavonoids in group D was the greatest (67% lost).

## Conclusion

This study examined the influences of different drying pretreatments of *D*. *erythrosora* leaves on total flavonoid contents, antioxidant activity, and flavonoid ingredients. The main conclusions were: a) The total flavonoids contents, antioxidant activities, and flavonoid ingredients in leaves with different drying pretreatments varied, and samples that were first dried in the shade then oven-dried at 75°C had the highest flavonoid content and strongest antioxidant activities. b) The pretreatment of rapid oven drying in a 75°C oven produced the greatest loss of flavonoids. The most successful pretreatment for conserving leaf flavonoids was the initial shade drying followed by oven drying at 75°C. Freezing and grinding in liquid nitrogen followed by filtering was also a useful technique.

## Supporting information

S1 Table Datas from the standard curve of rutin. (DOCX)
