# Supplementary material for: Effects of different pretreatments on flavonoids and antioxidant activity of Dryopteris erythrosora leave
Source: PLoS One. 2019 Jan 2;14(1):e0200174. doi: 10.1371/journal.pone.0200174 (PMC6314590; doi:10.1371/journal.pone.0200174)
Supplement: S1 Table — (DOCX) [file pone.0200174.s001.docx]

Table 1 Datas from the standard curve of Rutin

| Concentration (Rutin) | Absorbance/OD value |
| --- | --- |
| 0.025 | 0.129 |
| 0.04 | 0.296 |
| 0.05 | 0.411 |
| 0.075 | 0.684 |
| The fitted curve: Y=-0.14752+11.10566X; R=0.99996; SD=0.00367; p<0.0001 | |
